# Late Onset Hypercalcemia in an HIV Patient as a Manifestation of Immune Reconstitution Inflammatory Syndrome (IRIS)

**DOI:** 10.7759/cureus.57773

**Published:** 2024-04-07

**Authors:** Mallak Zatreh, Betty Drees, Jignesh Shah

**Affiliations:** 1 Internal Medicine, University of Missouri Kansas City School of Medicine, Kansas City, USA; 2 Endocrinology, Diabetes and Metabolism, University of Missouri Kansas City School of Medicine, Kansas City, USA; 3 Nephrology, University of Missouri Kansas City School of Medicine, Kansas City, USA

**Keywords:** acute kidney injury(aki)), granulomatous infection, mycobacterium avium intracellulare, hiv and aids, immune reconstruction inflammatory syndrome, calcitriol-mediated hypercalcemia

## Abstract

Hypercalcemia in human immunodeficiency virus (HIV) patients can be challenging due to various underlying mechanisms. 1,25- dihydroxycholecalciferol (1,25 (OH)2 vitamin D)-mediated hypercalcemia due to increased activity of extrarenal 1-alpha hydroxylase is one of the well-known mechanisms of hypercalcemia described in HIV patients. *Mycobacterium avium-intracellulare* (MAI) is a granulomatous disease that can cause hypercalcemia due to ectopic production of alpha -1 hydroxylase and result in increased levels of 1,25 (OH)2 vitamin D. Herein, we present a case of “late-onset” hypercalcemia in a patient with HIV/AIDS and MAI infection in the setting of suspected immune reconstitution inflammatory syndrome (IRIS). The hypercalcemia workup showed an inappropriately average level of 1,25 (OH)2 vitamin D while the rest of the workup was unrevealing. Unusually normal levels of vitamin D metabolites were the driving mechanism of hypercalcemia in this case. The late presentation of hypercalcemia was likely due to IRIS unmasking an underlying granulomatous infection, and this consideration led to the successful treatment of the patient with steroid administration. This case emphasizes the importance of considering IRIS in evaluating hypercalcemia that presents late in the course of granulomatous infections in HIV patients.

## Introduction

Hypercalcemia is associated with granulomatous disease. Both tuberculous and non-tuberculous *Mycobacterium* are associated with hypercalcemia. *Mycobacterium avium*-*intracellulare* (MAI) is a non-tuberculous *mycobacterium* that can cause hypercalcemia. The mechanism of hypercalcemia in granulomatous diseases is due to excessive production of 1,25 (OH)2 vitamin D by activated macrophages that produce alpha-1 hydroxylase enzyme [1]. One unique mechanism of hypercalcemia in HIV patients is through granuloma formation where the ectopic production of alpha -1 hydroxylase is unmasked after initiation of antiretroviral therapies (ART) as a manifestation of immune reconstitution inflammatory syndrome (IRIS) [2]. Although the mechanism of hypercalcemia in granulomatous disease is thought to be related to vitamin D dysregulation, this does not always correlate with vitamin D metabolite levels [3]. Below, we describe a disseminated MAI infection with severe hypercalcemia and an inappropriately normal 1,25-(OH)2 vitamin D without any other standard or uncommon causes of hypercalcemia.

## Case presentation

A 55-year-old male with a past medical history of HIV presented to the hospital with recurrent fever. Workup was significant for an undetectable CD4 count, a viral load of 3,000,000 copies/mL, and a positive acid-fast bacilli blood culture that eventually grew MAI. As a result, ethambutol, rifabutin, and azithromycin were started. Two weeks after the initiation of MAI treatment, antiretroviral therapy (ART) was started, and the patient was eventually discharged. Six months after initiating ART, the patient was rehospitalized with nausea, dizziness, and abdominal discomfort, which all began on the day of presentation. This patient denied any excessive milk intake, vitamin D, calcium, or multivitamin supplements; no recent thiazide diuretics or non-steroidal anti-inflammatory drugs use was noted. The infectious disease clinic notes indicated nonadherence to ART, with the most recent CD4 count after initiation of ART for at least 5 months being 64 cells/mm3 (reference 500-1500 cells/mm^3^). 

On physical examination, the patient was cachectic with a temperature of 36.3°C, blood pressure of 132/83 mmHg, heart rate of 83 beats/min, respiratory rate of 15/min, and an oxygen saturation of 98% on room air. The initial workup showed a low CD4 count and elevated levels of serum creatinine, calcium, and ionized calcium. A hypercalcemia workup revealed decreased parathyroid hormone (PTH) levels, a low level of 25-hydroxycalciferol, normal upper limit of 1,25 (OH)2 vitamin D level, and a normal PTH-related peptide (PTHrP). The laboratory work-up is summarized in Table 1. Urine calcium was high at 498 mg/24 hours. Further investigations, including serum protein electrophoresis, urine protein electrophoresis, Kappa and Lambda free light chain, and skeletal survey, did not show monoclonal gammopathy, elevated levels, or osteolytic lesion, respectively.

**Table 1:** Laboratory workup done during hospital admissions with hypercalcemia. The first admission workup reveals acute kidney injury (AKI), hypercalcemia, low PTH, and inappropriately normal 1,25 (OH)2 vitamin D. Hypercalcemia and AKI recurred during subsequent admissions.

	First hypercalcemia presentation	Second presentation	Third presentation	Reference range
Corrected calcium	14.2	12.9	16.3	8.4-10.4 mg/dL
Creatinine	4.26	4.06	4.6	0.8-1.2 mg/dL
Phosphorus	3.1	-	-	2.3-5.6 mg/dL
CD4 level	75	88	151	500-1500 cells/mm^3^
PTH	11.8	-	-	12-88 pg/mL
25 (OH) vitamin D	13.1	-	-	30-100 ng/mL
1,25 (OH)2 vitamin D	52	-	-	18-72 pg/mL
PTHrP	25	-	-	10-27 pg/mL

The kidney and abdominal ultrasound were normal except for nonobstructive renal calculi. The hypercalcemia was treated with intravenous fluids, calcitonin, and pamidronate, with normalization of serum calcium and improvement in the patient’s condition and symptoms within 48 hours. Renal function improved with hydration and improvement of hypercalcemia but did not return to normal, and serum creatinine remained at 2.7 mg/dL. The patient declined further workup and was discharged after normalization of serum calcium level. However, six weeks later, the patient presented again with similar symptoms and similar lab results of hypercalcemia and acute kidney injury, for which the patient received intravenous fluids and calcitonin. Additional workup was pursued during this admission. Computed tomography (CT) scan of the chest revealed a soft tissue density in the right perihilar region posterior to the superior vena cava, for which endobronchial ultrasound (EBUS) was recommended. Abdominal MRI showed diffuse lymphadenopathy. A kidney biopsy was performed, which showed calcium-related deposits with tubular injury, suggesting hypercalcemia-related kidney injury, with no granulomatous interstitial inflammation noted. The patient was discharged after further diagnostic evaluation was declined, with plans to follow up in the outpatient setting.

Eight weeks after discharge, the patient was seen in the clinic where serum calcium and creatinine were found to be elevated. As a result, the patient was admitted for the third time and treated with intravenous fluids and calcitonin. Given the patient's reluctance to undergo invasive workup, including biopsy, malignancy, particularly lymphoma, remained a differential diagnosis. Despite the absence of a definitive diagnosis, empiric treatment with glucocorticoids was implemented. 

**Figure 1:**
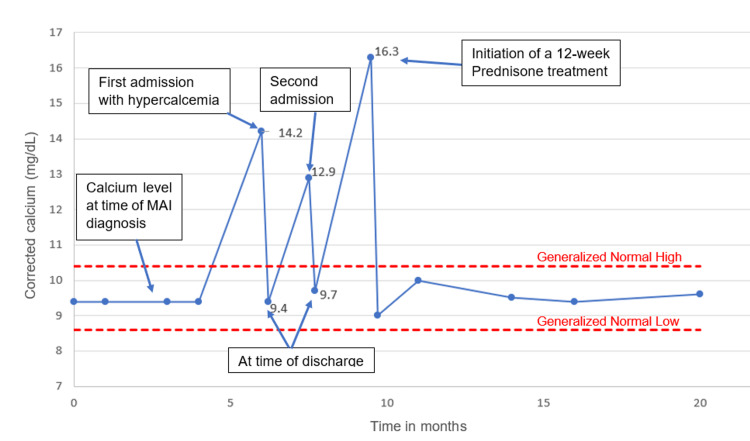
Trends in calcium levels before and after initiating prednisone therapy. Image Credits: Mallak Zatreh

After finishing a 12-week course of prednisone therapy with tapering, calcium levels stabilized between 9-10 mg/dL and serum creatinine between 1.2-1.4 mg/dL. Subsequent follow-up visits demonstrated sustained normocalcemia without the recurrence of hypercalcemia. Notably, even after discontinuation of prednisone, the patient has remained normocalcemic for nearly two years, as illustrated in Figure 1. 

## Discussion

Hypercalcemia in HIV-infected patients is rare but has been reported since the 1980s [4]. The reported mechanisms of hypercalcemia in HIV patients include increased osteoclastic activity due to infection (e.g., Cytomegalovirus infection), hypercalcemia of malignancy due to increased PTHrP (HTLV-1 infection causing T-cell Lymphoma), ectopic 1,25 (OH)2 vitamin D due to granulomatous diseases or lymphoma, and/or unknown mechanisms. 

The most severe cases of hypercalcemia in HIV are most often seen with lymphomas [5]. Hypercalcemia has been well-described in granulomatous diseases. Both tuberculous and non-tuberculous mycobacterial (NTM) infections have been associated with hypercalcemia. The exact mechanism of hypercalcemia in NTM infections is not fully understood. It may be related to excessive production of 1,25 (OH)2 vitamin D by activated macrophages, despite the high levels of serum calcium. Interestingly, elevated 1,25 (OH)2 vitamin D levels are not always seen in hypercalcemia associated with granulomatous disorders in the setting of HIV infections. A review of the literature yielded a few cases of MAI-associated hypercalcemia in the setting of normal 1,25 (OH)2 vitamin D levels [3,6,7]. Table 2 lists some reported cases of hypercalcemia in patients with granulomatous disease where 1,25 (OH)2 vitamin D levels are not elevated. Parsons et al. described one case of MAI infection in an immunocompetent patient without HIV or malignancy who had hypercalcemia with normal 1,25 (OH)2 vitamin D levels [6]. Another case report by Chatterjee et al. reported a patient with MAI infection and hypercalcemia in the presence of low 1,25 (OH)2 vitamin D levels [7]. This observation of hypercalcemia in the setting of granulomatous disease and normal 1,25 (OH)2 vitamin D levels has led to the suggestion of other alternate mechanisms of hypercalcemia in granulomatous diseases, such as elevated calcitonin levels, increased bone resorption, or increased levels of free 1,25 (OH)2 vitamin D secondary to hypoalbuminemia [3, 8].

**Table 2:** Reported cases of hypercalcemia in patients with granulomatous diseases where 1,25 (OH)2 vitamin D levels are not elevated, and no other explanation of hypercalcemia was found.

Author	Diagnosis	PTH	PTHrP	25-(OH) Vit D	1,25 (OH)2 Vit D
Parsons CE et al., 2017 [6]	MAI	Low	Normal	Normal	Normal: 27 pg/mL (Ref 18-78)
Chatterjee T et al., 2021 [7]	MAI	Low	Normal	Normal	Low: 11 pg/mL (Ref 18-78)
Shrayyef MZ et al., 2011 [3]	Sarcoidosis	Low	Normal	Normal	Normal
Kallas M et al., 2010 [8]	Granulomatous myositis	Low	Low	Normal	Normal: 64.9 pg/mL (Ref 22–79)
Nielsen CT et al., 2009 [9]	Mycobacterium Marinum	Low	Not measured	Low	Low: 17.49 pg/mL (Ref 25-75)

**Figure 2:**
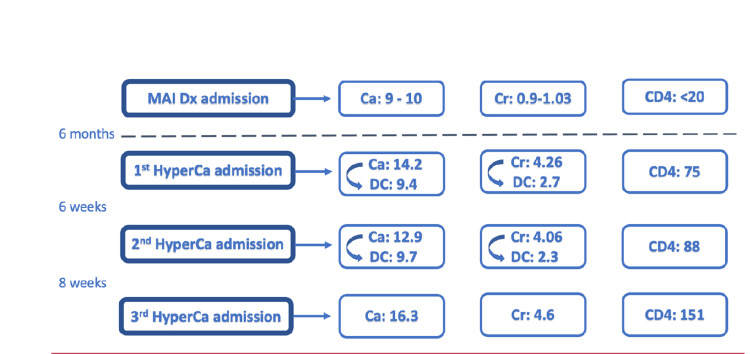
The temporal relationship between the onset of hypercalcemia and improvement in CD4 count, suggesting the possibility of IRIS presenting as hypercalcemia. MAI Dx: Mycobacterium Avium Intracellulare diagnosis. HyperCa: hypercalcemia. Ca: Calcium level (ref 8.4–10.4 mg/dL). Cr: serum creatinine level (ref 0.8-1.2 mg/dL). DC: Calcium/ Creatinine level at the time of discharge. CD4: CD4 count (ref 600-1500 cells/mm^3^). Image Credits: Mallak Zatreh

Another interesting aspect of this case is the timing of hypercalcemia in relation to MAI diagnosis. Our patient presented with hypercalcemia approximately six months after initiating MAI therapy and ART. This late onset of hypercalcemia could have been due to intermittent adherence to ART therapy, causing “late-onset” IRIS. After adherence to MAI and HIV treatment, the immune system was restored, and hypercalcemia developed. Figure 2 demonstrates the relationship between the onset of hypercalcemia and CD4 count improvement.

The suggested treatment of hypercalcemia in granulomatous disease, as well as in the setting of IRIS, is glucocorticoids. One of the mechanisms by which glucocorticoids reduce calcium levels in granulomatous diseases is through downregulating 1,25 (OH)2 vitamin D production by macrophages [10]. 

As shown in Figure 2, our patient had multiple recurrences of hypercalcemia, which in turn led to acute kidney injury (AKI) as well. This vicious cycle stopped after treatment with high-dose prednisone for 12 weeks, followed by tapering. 

The decision to forgo invasive workup posed significant challenges in determining the underlying cause of hypercalcemia. While malignancy, particularly lymphoma, remained a primary concern, the sustained normocalcemia without recurrence post-steroid discontinuation suggested a less likely malignant etiology. The absence of hypercalcemia recurrence for an extended period supports the notion of hypercalcemia secondary to IRIS as the most likely underlying etiology.

## Conclusions

HIV patients who are infected with granulomatous infections may or may not develop hypercalcemia. Hypercalcemia can develop early in the course of granulomatous infections or as a late manifestation, as shown in our case. The exact risk factors for hypercalcemia in these patients are still unknown. Genetic, environmental, and immunological factors have been suggested.

Although the known mechanism of hypercalcemia in HIV patients with granulomatous infections is due to increased activity of extrarenal 1-alpha hydroxylase with resultant increased levels of 1,25 (OH)2 vitamin D, there are a few case reports where hypercalcemia was found in the setting of normal or low 1,25 (OH)2 vitamin D and no other explanations for hypercalcemia. Although the exact mechanism for hypercalcemia in granulomatous infections with normal 1,25 (OH)2 vitamin D is still unknown, it is important to remain vigilant for this life-threatening complication and to treat patients appropriately.
